# Pulmonary Hypertension Secondary to Partial Anomalous Pulmonary Venous Return in an Elderly

**DOI:** 10.1155/2016/8609282

**Published:** 2016-02-23

**Authors:** Stefan Koester, Justin Z. Lee, Kwan S. Lee

**Affiliations:** University of Arizona, Tucson, AZ 85714, USA

## Abstract

*Background*. Partial anomalous pulmonary venous return (PAPVR) is an uncommon congenital abnormality, which may present in the adult population. It is often associated with sinus venosus defect (SVD). The diagnosis and therapy for this condition may be challenging.* Case Presentation*. We describe a case of an elderly woman who presented with NYHA Class IV dyspnea and was suspected to have symptomatic pulmonary hypertension. She was later found to have anomalous right upper pulmonary vein return to the superior vena cava and associated SVD with bidirectional shunting. Therapeutic options were discussed and medical management alone with aggressive diuresis and sildenafil was adopted. Follow-up visits revealed success in the planned medical therapy.* Conclusions*. PAPVR is a rare congenital condition that may present during late adulthood. The initial predominant left-to-right shunting associated with this anomaly may go undetected for years with the gradual development of pulmonary hypertension and right heart failure due to right heart volume overload. Awareness of the condition is important, as therapy is time-sensitive with early detection potentially leading to surgical therapy as a viable option.

## 1. Introduction

Partial anomalous pulmonary venous return (PAPVR) is an uncommon congenital abnormality, which may present in the adult population. We describe a case of an elderly woman who presented with symptomatic pulmonary hypertension and was found to have PAPVR associated with sinus venosus defect (SVD). We also discuss the diagnostic and therapeutic challenges encountered.

## 2. Case Presentation

A 77-year-old Hispanic lady with a history of asthma and elevated pulmonary pressures attributed to respiratory disease presented with increased dyspnea at rest and worsening lower extremity swelling over a week. Prior to this, she had been independent and was able to walk without symptoms. Her past medical history was otherwise significant for hypertension and hypothyroidism. Her home medications included albuterol, amlodipine, levothyroxine, loratadine, and metoprolol. She was not a smoker and consumed no alcohol. Systems review was otherwise unremarkable.

Physical exam showed an irregular rhythm with a rate in the 130 s. Blood pressure was 147/88 mmHg; she was afebrile, tachypneic with O_2_ sats. of 93%. She was a thin, elderly woman who was alert and cooperative. Her JVP was elevated at 15 cm H_2_O. She had mild, diffuse wheezing bilaterally with bibasal fine, inspiratory crackles. Heart sounds were present with fixed splitting of the second heart sounds and a 2/6 pan-systolic murmur in the right parasternal base. Abdomen was soft and nontender. She had 2+ bilateral lower extremity edema.

A 12-lead ECG showed atrial fibrillation with a rate of 133 bpm. A chest X-ray showed cardiomegaly and prominence of the pulmonary arteries with a small right pleural effusion.

Lab tests including a complete blood count and complete-metabolic panel were unremarkable. Troponin was borderline elevated and B-type Natriuretic Peptide was moderately elevated at 626 pg/mL. TSH was normal.

**Figure 1:**
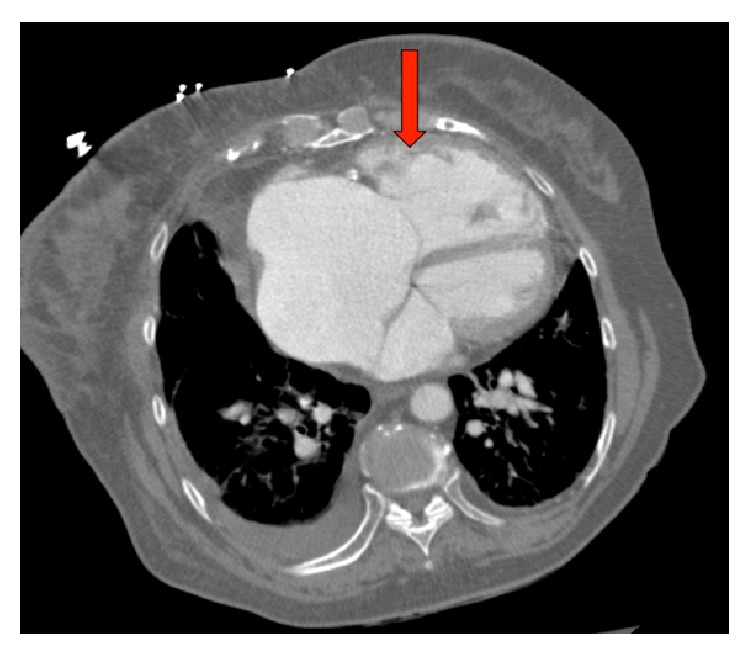
CT-scan showing right ventricular enlargement (red arrow).

**Figure 2:**
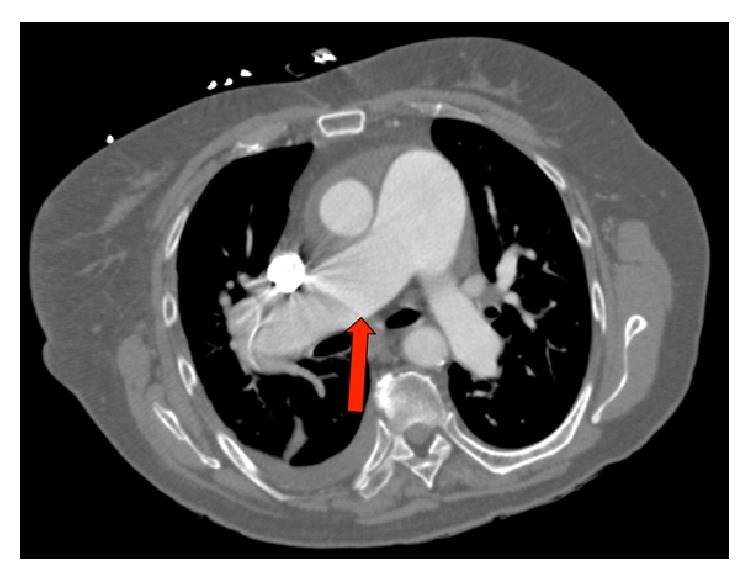
CT-scan showing enlarged pulmonary artery (red arrow).

**Figure 3:**
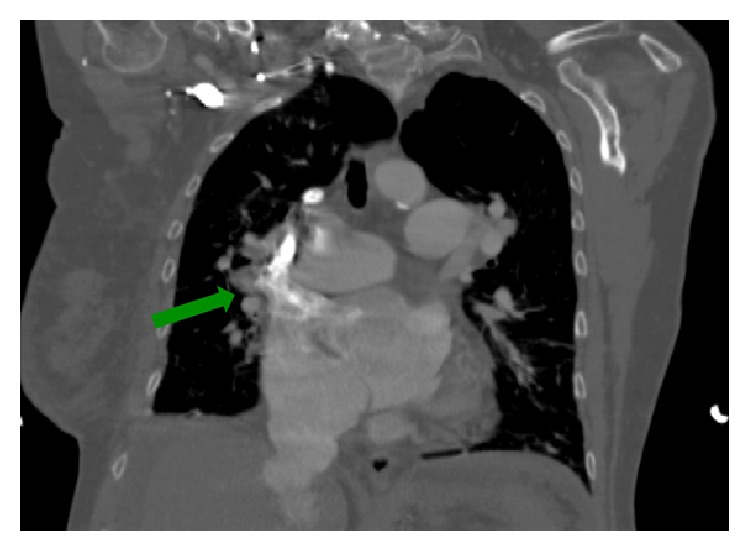
CT-scan showing anomalous right upper pulmonary venous return to superior vena cava (green arrow).

A chest CT with pulmonary angiography showed no pulmonary emboli. There was right heart cardiomegaly and dilation of the main pulmonary artery with calcifications seen in the proximal pulmonary arteries (Figures 1 and 2). Small bilateral pleural effusions were noted. A possible anomalous right upper pulmonary vein to lateral superior vena cava just above the right atrium to superior vena cava junction was also seen (Figure 3), with a possible associated interatrial septal defect. Compression ultrasonography with Doppler flow study revealed a nonocclusive deep venous thrombosis that was seen in the mid right superficial femoral vein.

**Figure 4:**
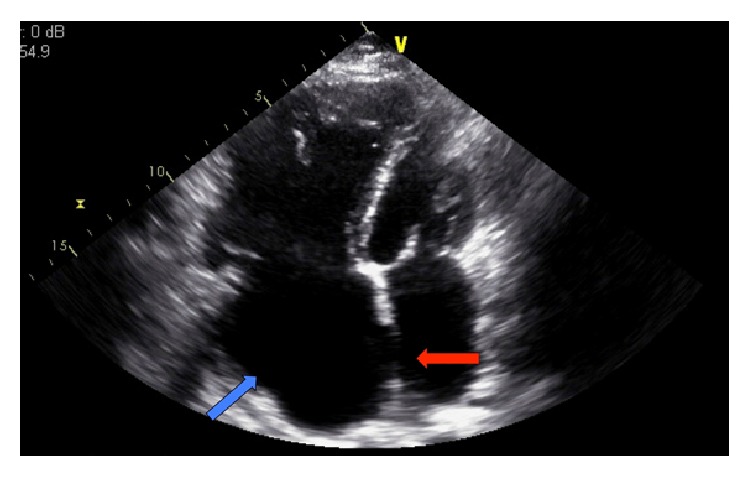
Echocardiography showing enlarged right ventricle and right atrium (blue arrow) with sinus venosus defect (red arrow).

Transthoracic echo showed left ventricular ejection fraction (LVEF) of 45%. The right heart was severely dilated with severe reduction in systolic function associated with systolic and diastolic ventricular septal flattening (Figure 4). Moderate mitral regurgitation and severe tricuspid regurgitation were noted with right ventricular systolic pressure (RVSP) estimated at 50 mmHg + CVP. Her IVC was dilated with minimal inspiratory change. Severe, resting right-to-left shunting was seen with saline bubble study.

**Figure 5:**
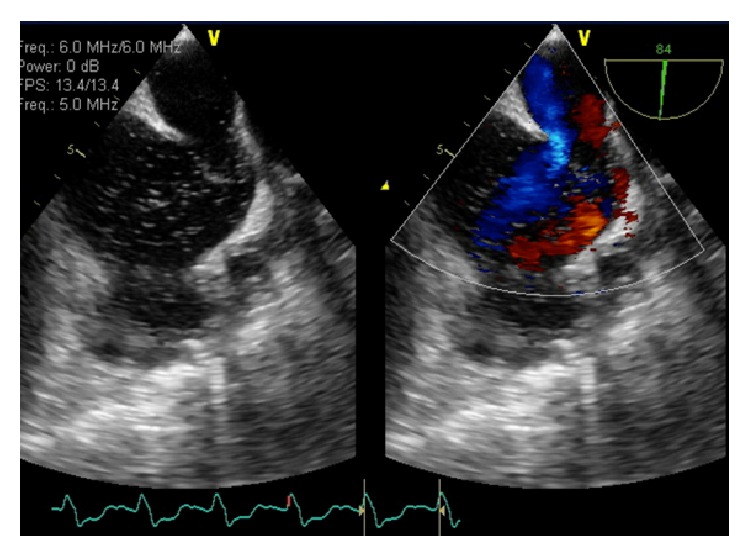
Color Doppler displaying sinus venosus defect.

Transesophageal echocardiography (TEE) guided cardioversion was performed. The TEE confirmed a sinus venosus (SV) atrial septal defect measuring 3.1 cm in maximum diameter (Figure 5). No left atrial appendage thrombus was seen. The right upper anomalous pulmonary venous return to the SVC was seen with no additional anomalous venous drainage. She was successfully cardioverted. Following atrial fibrillation cardioversion, her LVEF normalized with persistent right heart dilatation.

Right heart cardiac catheterization was performed with inhaled nitric oxide reactivity testing after several additional days of diuresis, initiation of digoxin, and the commencement of oral sildenafil. This showed a normal CVP of 2 mmHg, left atrial pressure of 6 mmHg, high-flow pulmonary hypertension of 74/15, and mean 37 mmHg. Pulmonary cardiac output was 12.5 L/min with systemic cardiac output of 8.7 L/min, resulting in a *Qp*/*Qs* of 1.4. Effective cardiac output was 5.1 L/min. Pulmonary vascular resistance was minimally elevated at 2.3 Wood units. Bidirectional shunting was present with predominant left to right shunt of 60% and right to left shunt of 40%. She had no significant response with inhaled nitric oxide and her systemic oxygen saturation corrected on a 100% O_2_ nonrebreather.

Initial anticoagulation with iv heparin and warfarin was initiated for the DVT, which also prepared her for TEE-guided cardioversion. Rate control of her atrial fibrillation was initially achieved with oral digoxin. Beta-blockers and nondihydropyridine calcium channel blockers were not used secondary to her severe right heart failure. Oral sildenafil was initiated at this point at the recommendation of the pulmonary hypertension service. Aggressive IV diuresis was initiated.

After several days of initial stabilization, she was then scheduled for a TEE. This showed no left atrial appendage thrombus, and she was loaded with iv amiodarone. Elective biphasic cardioversion of her atrial fibrillation was performed. Two liters/min of home oxygen supplementation was started.

She improved over the course of therapy from NYHA Class IV symptoms to NYHA Class II symptoms by discharge. On follow-up in pulmonary hypertension clinic a month later, she remained symptomatically improved with good INR follow-up and atrial fibrillation free.

## 3. Discussion

We describe a case of late clinical presentation of symptomatic pulmonary hypertension secondary to PAPVR and a SVD with bidirectional shunting. PAPVC is a rare congenital heart disease, with a prevalence of 0.1 to 0.2% in the adult population [1, 2]. Anomalous right sided pulmonary veins could return to the right atrium, superior vena cava, inferior vena cava, azygos vein, hepatic vein, or portal vein. Anomalous left sided pulmonary veins might drain into the innominate vein, coronary sinus, and hemiazygos vein. Studies based on the pediatric population identified the most common form of PAPVR to be the right upper pulmonary vein connecting to the SVC which is most often associated with a SVD [3]. Interestingly, retrospective reviews of adults receiving CT imaging identified that only half of the anomalies were right sided, with the only case of right upper lobe PAPVR being associated with atrial septal defect [2]. This may be an indication that the pediatric and adult populations with PAPVR may be significantly different. Our patient was found to have PAPVR associated with right upper anomalous pulmonary venous return to the SVC with associated SVD.

In PAPVR, the persistent systemic venous connection acts similarly to a left-to-right shunt, where a portion of the right ventricular output is continuously recirculated and oxygenated blood is returned to the right heart without traveling to the systemic circulation. The initial predominance of left-to-right shunting causes the condition to be clinically undetected. Over time, the increase in pulmonary blood flow can lead to progressive remodelling of the pulmonary circulation and increased pulmonary vascular resistance, leading to pulmonary arterial hypertension [4]. This leads to gradual negative right heart remodelling occurring over the years due to right heart volume overload. As volume related pulmonary hypertension develops, severe tricuspid regurgitation often occurs associated with right atrial arrhythmias which may lead to sudden clinical decompensation. Over time, worse right failure occurs and may lead to reversal of the bidirectional shunting into predominant right-to-left shunting, systemic cyanosis and Eisenmenger's syndrome.

The diagnosis of PAPVC is challenging. The typical presenting clinical features such as shortness of breath, right heart failure, and pulmonary hypertension are not specific to PAPVC. Therefore, patients may be misdiagnosed initially as having primary pulmonary hypertension [5]. In our patient, diagnosis was made after CT pulmonary angiogram was performed to exclude thromboembolic disease (Figures 1, 2, and 3). This is similar to other cases where PAPVR was diagnosed following CT-angiogram to exclude pulmonary embolism [6, 7].

Awareness of the condition is important, as early recognition can lead to successful surgical correction which involves surgical redirection of the anomalous vein into the left atrium [8]. In our patient, her severe pulmonary arterial hypertension and elevated pulmonary vascular resistance preclude her as a good surgical candidate. It is not likely that surgery is going to alter the disease course, as the extensive vascular remodelling is unlikely to be reversible. Catheter embolization of the anomalous vein was also considered. However, this was not a viable option in our patient as there was no concomitant connection from the anomalous vein to the left atrium, which can accommodate the venous drainage after the anomalous vein has been embolized [9]. In our patient, given her high surgical risk, medical therapy alone was adopted. There have been small retrospective and observational studies of patients with pulmonary arterial hypertension secondary to congenital heart diseases, including PAPVR, having clinical and hemodynamic improvements with prostaglandins, phosphodiesterase inhibitors, and bosentan [10]. However, close monitoring of response to treatment is important in the event that the pulmonary hypertension progresses.

In conclusion, PAPVR is a rare congenital condition that may present during late adulthood. The predominant left-to-right shunting associated with this anomaly can go undetected for years with the gradual development of pulmonary hypertension and right heart failure due to right heart volume overload. There should be a high index of suspicion, especially in patients with unexplained pulmonary hypertension. Awareness of the condition is important, as therapy is time-sensitive with early detection potentially leading to surgical therapy as a viable option.
