# Corrigendum to “Pulmonary Hypertension Secondary to Partial Anomalous Pulmonary Venous Return in an Elderly Patient”

**DOI:** 10.1155/2019/2347179

**Published:** 2019-11-21

**Authors:** Stefan Koester, Justin Z. Lee, Kwan S. Lee

**Affiliations:** University of Arizona, Tucson, AZ 85714, USA

In the article titled “Pulmonary Hypertension Secondary to Partial Anomalous Pulmonary Venous Return in an Elderly” [1], there was an error in the article's title where the word “Patient” should be added at the end of it. The updated title is shown above.

## References

[1] Koester S., Lee J. Z., Lee K. S. (2016). Pulmonary hypertension secondary to partial anomalous pulmonary venous return in an elderly. *Case Reports in Cardiology*.

